# Does advancing male age influence the expression levels and localisation patterns of phospholipase C zeta (PLCζ) in human sperm?

**DOI:** 10.1038/srep27543

**Published:** 2016-06-08

**Authors:** Marc Yeste, Celine Jones, Siti Nornadhirah Amdani, Suseela Yelumalai, Ginny Mounce, Sarah J. Martins da Silva, Tim Child, Kevin Coward

**Affiliations:** 1 Nuffield Department of Obstetrics and Gynaecology, University of Oxford, Level 3, Women’s Centre, John Radcliffe Hospital, Headington, Oxford, OX3 9DU, United Kingdom; 2 PAPRSB Institute of Health Sciences, Universiti Brunei Darussalam, Jalan Tungku Link, Gadong, Brunei Darussalam; 3 Medical Research Institute, University of Dundee, Ninewells Hospital and Medical School, Dundee, DD1 9SY, UK; 4 Assisted Conception Unit, NHS Tayside, Ninewells Hospital, Dundee, DD1 9SY, UK; 5 Oxford Fertility, Institute of Reproductive Sciences, Oxford Business Park North, Alec Issigonis Way, Oxford, OX4 2HW.

## Abstract

Socio-economic factors have led to an increasing trend for couples to delay parenthood. However, advancing age exerts detrimental effects upon gametes which can have serious consequences upon embryo viability. While such effects are well documented for the oocyte, relatively little is known with regard to the sperm. One fundamental role of sperm is to activate the oocyte at fertilisation, a process initiated by phospholipase C zeta (PLCζ), a sperm-specific protein. While PLCζ deficiency can lead to oocyte activation deficiency and infertility, it is currently unknown whether the expression or function of PLCζ is compromised by advancing male age. Here, we evaluate sperm motility and the proportion of sperm expressing PLCζ in 71 males (22–54 years; 44 fertile controls and 27 infertile patients), along with total levels and localisation patterns of PLCζ within the sperm head. Three different statistical approaches were deployed with male age considered both as a categorical and a continuous factor. While progressive motility was negatively correlated with male age, all three statistical models concurred that no PLCζ–related parameter was associated with male age, suggesting that advancing male age is unlikely to cause problems in terms of the sperm’s fundamental ability to activate an oocyte.

Infertility affects 1 in 7 couples and is defined as the inability of a couple to achieve pregnancy after one year of unprotected intercourse^1^. Considered as a disease by the World Health Organisation (WHO)^2^, infertility is known to arise from multi-factorial origins. Between 25 and 30% of cases relate to a male factor, while 20–35% relates to a female factor, and 25–40% arise from a combination of both male and female factors. Worryingly, 10–25% of cases remain idiopathic/unexplained (reviewed in ref. 3).

Male factor infertility is frequently associated with abnormal semen quality parameters such as low sperm concentration (oligozoospermia), poor motility (asthenozoospermia), abnormal morphology (teratozoospermia) or even the complete absence of sperm (azoospermia). Such problems can be attributed to spermatogenic deficiencies and/or abnormal epididymal maturation, and may arise from either genetic or extrinsic factors^4^. In this context, advancing male age has been reported to be associated with a gradual decline in sperm quality, which may result in sub-fertility^5^,^6^. This reduction in sperm quality not only affects conventional parameters, such as semen volume and sperm motility^7^,^8^,^9^, but is also related to increased proportions of sperm with either fragmented DNA^10^,^11^,^12^,^13^, or chromosomal defects^14^,^15^.

Upon ovulation, oocytes are held in metaphase-II arrest and can only complete meiosis-II when activated by the fertilizing sperm^16^. A mounting body of evidence from both basic and clinical research now provides clear support for phospholipase C zeta 1 (PLCζ) as the sperm-borne protein factor responsible for activating the oocyte upon gamete fusion^17^,^18^,^19^. Following diffusion into the ooplasm, PLCζ triggers a series of intracellular calcium oscillations that subsequently drives a cascade of biological events, including cortical granule exocytosis, prevention of polyspermy, polar body extrusion, cytoskeletal rearrangements and the formation of pronuclei^17^,^18^,^19^,^20^,^21^.

Over recent years, a series of clinical studies have clearly related PLCζ deficiency to male infertility. For example, abnormal expression levels or genetic mutations may lead to oocyte activation deficiency (OAD) and total fertilisation failure (TFF)^22^,^23^,^24^,^25^,^26^,^27^. Furthermore, the proportions of sperm exhibiting PLCζ in a semen sample are correlated with fertilisation outcome following intracytoplasmic injection (ICSI) but not following *in vitro* fertilisation (IVF)^28^. In addition, although seven different PLCζ-localisation patterns have been identified in human sperm (acrosomal; post-acrosomal; equatorial; acrosomal and post-acrosomal; acrosomal and equatorial; post-acrosomal and equatorial; acrosomal, post-acrosomal and equatorial)^29^, only the specific localisation of PLCζ in the equatorial and post-acrosomal regions are correlated with fertilisation rates following ICSI^28^. Moreover, while genetic causes, such as those underlying globozoospermia, may result in sperm which are devoid of PLCζ^25^,^26^,^30^, the potential effects of other factors, including male age, have received far less attention^31^. Such considerations are becoming very relevant because, for various socio-economic factors, the age of fatherhood is progressively increasing. Indeed, the proportion of men aged 35–55 years of age and fathering a child has increased by approximately 15% over the last decade^32^.

Against this background, the present study sought to determine whether paternal age exerts impact upon the expression or localisation of PLCζ in human semen samples, and to determine whether the influence of age differs between fertile controls and infertile patients.

## Results

### Differences between age groups and between fertile controls and infertile patients in the proportions, total levels and localisation patterns of PLCζ

**Figure 1:**
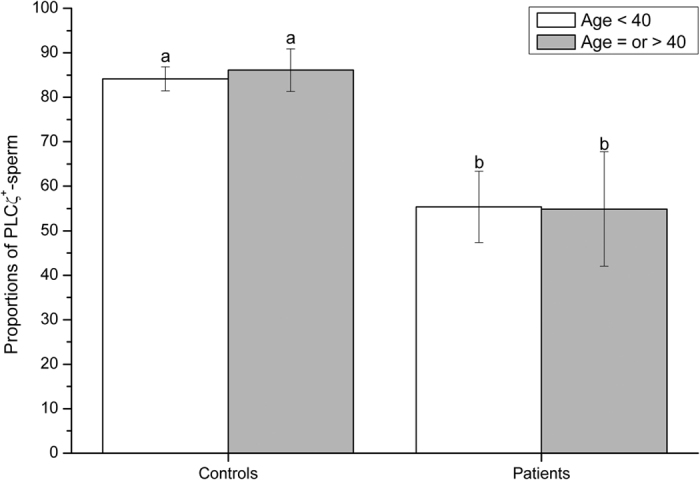
Proportions of sperm exhibiting PLCζ (mean ± SEM) in fertile controls and infertile patients younger than 40 years of age, or equal to and older than 40 years of age. Different letters in superscript (**a,b**) denote significant (*P* < 0.05) differences between controls, patients and age groups. Fertile controls presented significantly higher proportions of sperm exhibiting PLCζ than infertile patients, but no significant differences were found in relation to male age.

**Figure 2:**
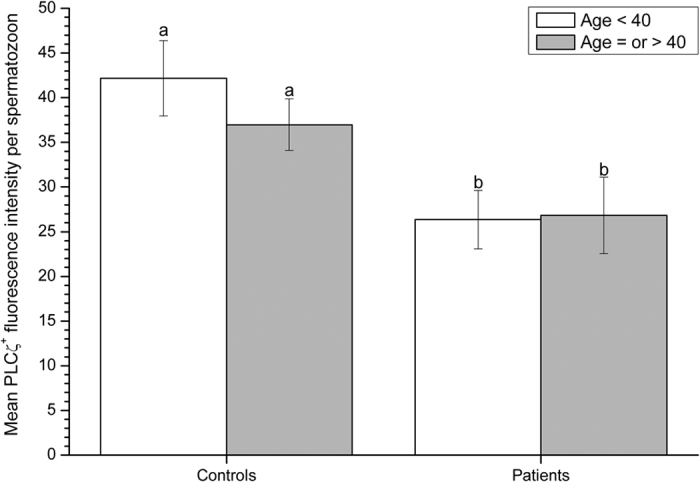
Total levels of PLCζ per spermatozoon (fluorescence intensity, arbitrary units), as mean ± SEM, in fertile controls and infertile patients less than 40 years of age, or equal to and older than 40 years of age. Different letters in superscript (**a,b**) denote significant (*P* < 0.05) differences between controls, patients and age groups. Fertile controls presented significantly higher proportions of sperm exhibiting PLCζ than patients, but no significant differences were found in relation to male age.

When age was considered as a categorical factor, no significant (*P* > 0.05) differences were detected between the two age groups (i.e.  <40 and ≥40) within either fertile control or infertile patient groups (Fig. 1). In contrast, the proportions (%) of sperm exhibiting PLCζ were significantly (*P* < 0.05) higher in controls than in patients, for both age groups. No interaction (*P* > 0.05) between the classification of samples (fertile controls or infertile patients) and age groups were observed and, thus, the effects of age on the proportions of sperm exhibiting PLCζ were similar in control and patient groups. Similar findings were observed for the total levels of PLCζ, in which fertile controls presented significantly (*P* = 0.01) higher levels of PLCζ than infertile patients, but without significant differences (*P* > 0.05) between the two age groups (Fig. 2).

The proportion (%) of sperm exhibiting PLCζ in acrosomal (A), post-acrosomal (PA), acrosomal + post-acrosomal (A + PA), acrosomal + equatorial (A + E), post-acrosomal + equatorial (PA + E) and acrosomal + post-acrosomal + equatorial (A + PA + E) localisation patterns did not differ significantly, either between fertile controls and infertile patients, or between the two age groups (Table 1). In contrast, the proportion (%) of sperm exhibiting PLCζ in the equatorial region only were significantly higher, and those devoid of PLCζ significantly (*P* < 0.05) lower, in fertile controls than in infertile patients. In spite of such differences, there were no significant differences between the age groups tested.

### Correlation of male age with proportions, total levels and localisation patterns of PLCζ

None of the parameters evaluated for PLCζ (proportions, total levels or localisation patterns), either in fertile controls or infertile patients, was significantly correlated with male age (Table 2). The same result was obtained when samples from fertile controls and infertile patients were analysed collectively as one common group.

### Clustering of data into fertile controls and infertile patients, and age groups

All parameters used to evaluate PLCζ (proportions, total levels, and localisation patterns) were also used for additional cluster analysis. The optimal number of clusters was two and the descriptive parameters used for these two clusters are shown in Table 3. When logistic regression models were used to evaluate the effects of age (considered as both continuous and categorical factors), it was apparent that male age (χ^2^ Wald = 0.05; *P* > 0.05) did not determine the pertinence of cases to any specific cluster. In contrast, the male pertinence to either fertile control or infertile patient groups was included as a factor in the model (χ^2^ Wald = 11.09; *P* < 0.05), which indicated that pertinence of a given case to a specific cluster relied on whether the case was a fertile control or an infertile patient.

### Relationship between age and other sperm quality parameters

Male age was significantly (*P* < 0.05) associated with total sperm motile count and the proportion of sperm with progressive motility. This was not only observed when male age was considered as a categorical variable, but also when correlations were calculated with male age being considered as a continuous variable (Table 4). When correlations were calculated from individuals belonging to controls or patients groups, the age was correlated to motile count and progressive sperm motility.

## Discussion

Although advancing parental age is recognised as a risk factor for sub-fertility, the age of fatherhood has been steadily increasing over the last few decades, particularly in Western countries^32^. Thus far, the bulk of research attention has focused upon the consequences of advancing maternal age and the potential implications for mother and child^33^,^34^. In comparison, far less focus has been directed towards the potential effect of paternal age^5^. Within this limited body of research, previous work has established that advancing male age results in a steady reduction in sperm quality, increased proportions of sperm with fragmented DNA and an increased risk of infertility^6^,^9^,^10^,^13^,^35^. However, it remains to be determined whether male age has any other effects upon relevant sperm proteins, such as PLCζ.

One fundamental role of the sperm is to activate the oocyte upon gamete fusion at fertilisation. This vital signalling mechanism is initiated and regulated by PLCζ, a sperm-specific protein, and a number of previous publications have associated PLCζ-deficiency with oocyte activation deficiency or total fertilisation failure^22^,^23^,^25^,^26^,^28^,^36^. While biochemical and clinical evidence continues to accrue with regard to the implicit association between PLCζ and successful oocyte activation^3^,^17^, very little is known as to how PLCζ-deficiency arises in the first place. The present study firstly confirmed that infertile patients presented lower proportions of sperm exhibiting PLCζ, as reported previously^28^,^36^, but secondly, failed to find any correlation between male age and any of the PLCζ-related parameters tested (i.e. total levels, localisation patterns, or the proportions of sperm exhibiting PLCζ). We arrived at the same conclusion using three distinct and independent statistical approaches (ANOVA, correlation, cluster analysis and further logistic regression). In addition, the absence of age effects upon PLCζ-related parameters was not only observed when samples were separated as fertile controls and infertile patients, but also when these two groups were analysed collectively as one larger cohort.

Two of the three statistical approaches employed (ANOVA, and cluster analysis followed by logistic regression) considered male age as a categorical factor. The cut-off value for classifying patients into two different age groups was assigned to 40 years of age, concurring with previous studies that reported an apparent reduction in sperm quality upon reaching this particular chronological milestone^37^,^38^. In addition, considering male age as a categorical or as a continuous factor yielded the same results, thus providing further justification to support our cut-off age value. Finally, clustering the samples according to proportions, total levels and PLCζ-localisation patterns was not dependent on male age, either in fertile controls or in infertile patients.

In contrast to PLCζ-related parameters, male age was negatively correlated with motile sperm count and the proportion of sperm exhibiting progressive motility. These results are in agreement with earlier studies investigating the effects of age upon sperm^37^,^39^. The fact that none of the three statistical approaches adopted to examine the effects of age upon PLCζ allowed us to distinguish between fertile controls and infertile patients not only made our current study comparable with previous reports, but also allowed us to exclude any interaction between male age and fertility status. In this regard, it is worth mentioning that Plastira *et al.*^10^ reported that while male age was negatively associated with sperm motility, morphology and DNA integrity in an infertile patient group, such age-effects were not evident in a fertile control group. In contrast to this study, the present work found a clear reduction of sperm motility with advancing age in both control and patient groups.

As mentioned earlier, increasing male age is related to reductions in semen volume, sperm motility and normal morphology, along with an increased likelihood of sperm DNA fragmentation^6^,^7^,^8^,^9^,^10^,^11^,^13^. It has been suggested that such reductions in sperm quality could arise from deterioration of the testicular tissue architecture which involves thickening of the basal membrane in seminiferous tubuli, reduction of the seminiferous epithelium and in the number of both Sertoli and Leydig cells, along with compromised testicular vascularisation^40^. An additional explanation could be related to changes in composition of seminal plasma occurring with advanced age^41^.

Although the precise mechanisms underlying the observed differences between fertile controls and infertile patients in terms of the proportion, total levels, and localisation pattern of PLCζ found here, and in previous studies^28^,^36^, are not yet fully understood, previous research has identified that genetic factors can underlie PLCζ deficiency, at least in some patients. For example, globozoospermia, an extreme case of teratozoospermia in which sperm can be totally devoid of PLCζ^25^,^30^,^42^, is known to have a genetic cause^43^. In addition, two point mutations in the coding region of the PLCζ gene have been found in a non-globozoospermic, infertile patient. These mutations affected the X and Y domains of PLCζ (substitution of histidine for leucine at residue 233 [H233L], and substitution of histidine for proline at residue 398 [H398P]), and were directly responsible for oocyte activation failure^23^,^25^,^26^. In the present work, we have shown that proportions, localisation patterns and total PLCζ levels are independent from male age. Since genetic factors have only been related to a very small number of patients thus far, more research is warranted to address which other factors might underlie the differences between fertile controls and infertile patients observed here and in other previous studies^27^,^28^,^36^.

The effect of paternal age on fertility outcome is far less consistent than those related to sperm quality. Indeed, while some studies indicate a positive correlation between age and infertility risk in men^9^,^35^, it is prudent to consider that other factors could potentially confound our ability to evaluate the impact of paternal age upon fertilising ability. For example, maternal age, and a reduction in the frequency of intercourse with advancing age^38^,^44^. In fact, previous studies have failed to identify an association between male age and fertilisation outcomes following *in vitro* fertilisation (IVF), even when donated oocytes were used, and therefore, any maternal effect had been removed^45^,^46^,^47^. In addition, the fact that Spandofer *et al.*^48^ failed to detect any significant effect of male age upon fertilisation outcomes following ICSI, is consistent with our present results, since proportions of sperm exhibiting PLCζ are known to be positively correlated with fertilisation rates^28^. Furthermore, confounding factors and covariates that are associated with age, such as vascular disease, obesity and factors related to lifestyle, may also mask the actual contribution of advancing age to the observed decline in sperm quality^49^,^50^,^51^,^52^.

Since the contribution of sperm to events occurring post-fertilisation is not restricted to PLCζ, the findings reported herein do not exclude the possibility that advancing male age could influence other important sperm factors. For example, sperm chromatin integrity, which has been linked to total fertilisation failure and miscarriage, is related to male age^12^,^53^,^54^,^55^.

In conclusion, the present study reports, for the first time, that the proportions, total levels and localisation patterns of PLCζ in human ejaculated sperm are not associated with male age. Consequently, while ageing is related to a clear reduction in sperm quality, it appears that the aging process does not affect all sperm proteins, but rather acts upon specific parameters. Therefore, patients with reduced sperm quality due to age may be advised to use IVF/ICSI, since advancing male age is unlikely to cause problems in terms of the sperm’s fundamental ability to activate an oocyte and initiate the process of embryogenesis. Of course, this does not exclude that these processes may be influenced by the oocyte quality, and in particular the viability of oocyte proteins that might interact with PLCζ during gamete fusion.

## Methods

### Ethics

Males were recruited from the Oxford Fertility Unit (OFU; Oxford, UK) and from the Assisted Conception Unit (ACU; Ninewells Hospital, Dundee, UK) with approval from the National Research Ethics Service (South Central Oxford Committee C; Reference number: 10/H0606/65) and the East of Scotland Research Ethics Service (EoSRES) (REC 1; Reference number: 13/ES/0091), respectively. Informed consent was obtained from all subjects involved in this study and all methods described in this section were carried out in accordance with the guidelines approved by the two Ethical Committees.

### Sperm samples

Semen samples were provided following at least three days of abstinence. A total of 71 males were recruited into this study, ranging from 22 to 54 years of age (age, mean ± standard error of the mean, SEM: 36.4 ± 0.7). All males were classified into either a fertile control group or an infertile patient group, as described by Yelumalai *et al.*^28^. The control group (n = 44; age, mean ± SEM: 35.8 ± 1.0) included (i) men with normal semen analysis parameters that were able to successfully fertilise an oocyte following assisted reproductive technology (ART), (ii) men with no history of infertility or oocyte activation deficiency, and (iii) men who had previously fathered children via natural conception. These clinical data were provided by the respective clinical uit providing the sample. All other samples were classified as infertile patients (n = 27; age, mean ± SEM: 37.4 ± 1.1 years) based upon sperm quality analysis, including total motile count, the proportion of sperm exhibiting progressive motility, sperm concentration (sperm·mL^−1^), and morphological abnormalities (e.g. sperm presenting pin-shaped or round heads). A history of total fertilisation failure following IVF and/or ICSI was also used as a criterion to classify samples into the infertile patient group^28^.

### Preparation of sperm samples

Semen volume, pH, viscosity, sperm motility and sperm concentration were evaluated upon arrival in our laboratory, and were then subjected to density gradient washing (DGW) using PureSperm™ (PureSperm™ 40/80, Nidacon International AB, Gothenburg, Sweden), as described previously^56^. In brief, 2 mL of 80% PureSperm medium was layered onto the bottom of a 15 mL tube and overlaid with 2 mL of 40% PureSperm medium. Subsequently, 1.5 mL of liquefied semen sample was layered on top of the 40% PureSperm medium and samples centrifuged at 300×*g* (room temperature) for 20 min. Following the centrifugation step, most of the supernatant was discarded and approximately 0.5 mL of pellet remained at the bottom of the tube. This pellet was then transferred to a clean 15-mL tube containing 5 mL of PureSperm™ Wash medium (Nidacon International AB). This mixture was subsequently centrifuged at 500 × *g* (room temperature) for 10 min. The pellet was resuspended in 1 mL of PBS to evaluate sperm motility and 200 μL was used for PLCζ analysis. This latter aliquot was centrifuged at 800 × *g* (room temperature) for three min and the supernatant discarded without disturbing the remaining pellet. This pellet was resuspended with 100 μL 4% paraformaldehyde (Sigma-Aldrich; Gillingham, Dorset, UK) and incubated at room temperature for 10 min in order to fix sperm cells. Following a final centrifugation step, and removal of paraformaldehyde, the sperm pellet was re-suspended and stored in 200 μL PBS at 4 °C to await immunofluorescent analyses.

### Evaluation of sperm quality

Sperm concentration and motility were assessed before and after DGW, as described in ref. 28, and according to WHO guidelines^57^. For the evaluation of sperm concentration, samples were diluted with sterile water (at a ratio of 1/20) and counted using a Neubauer chamber (Paul Marienfeld GmbH & Co. KG; Lauda-Königshofen, Germany). Two separate counts per sample were carried out.

Sperm motility was also evaluated in two independent replicates, in accordance with the latest WHO guidelines^57^. This analysis was performed taking into account the proportion of sperm exhibiting progressive sperm motility and the motile count, which represented the number of sperm exhibiting progressive motility.

### Purification and specificity of the PLCζ antibody

Anti-human PLCζ antibody was synthesised by Cova-Lab (Cambridge, UK) against two immunogenic peptides identified in the human PLCζ amino acid sequence (C-RESKSYFNPSNIKE-coNH_2_ and C-ETHERKGSDKRGDN; Accession Number: AF532185). Before use, the crude antibody was purified with a SulfoLink Kit (Pierce Biotechnology, Rockford, USA), and the two original immunogenic peptides. Specificity of the PLCζ antibody was confirmed by competitive pre-incubation studies with excess peptides (data not shown), following the procedure described in ref. 29. The PLCζ antibody was proven to be specific since no immunostaining was observed when samples were incubated with antibody in the presence of blocking peptides. It is worth noting that the same antibody has been used in several other studies arising from this laboratory, and others, and has provided a consistent pattern of immunostaining throughout^23^,^25^,^28^,^29^,^36^,^56^.

### Immunofluorescent detection of PLCζ

Sperm samples were immunostained for PLCζ using the protocol described by Grasa *et al.*^29^. First, a 100-μL drop of fixed sperm was placed onto a slide pre-coated with 0.01% Poly-L-Lysine and permeabilised with 0.5% Triton X-100 in PBS (Sigma Aldrich) overnight. Slides were subsequently incubated with 3% bovine serum albumin (BSA, Sigma-Aldrich) at room temperature for one hour, and then with 100 μL of anti-PLCζ antibody (25 μg·mL^−1^; Cova-Lab, UK) diluted in 0.05% BSA at 4 °C and overnight. After this step, samples were washed three times with PBS and then incubated with 5 μg·mL^−1^ of a secondary goat anti-rabbit antibody conjugated with Alexa Fluor 488 (Invitrogen, Paisley, UK) at room temperature for 1 hour. After re-washing the slides with PBS a total of three times, 3 μL of Vectashield H-1200 mounting medium containing 4′-6′-Diamidini-2-phenylindole (DAPI) (Vector, UK) was added onto the top of the sample prior to covering with 20 mm × 20 mm glass cover slips.

Evaluation of samples was performed under a fluorescence microscope (Eclipse 80i, Nikon UK Ltd., Kingston upon Thames, UK) at 400× magnification. Since the excitation wavelength for the secondary antibody was 488 nm, a fluorescein isothiocyanate filter (FITC; Excitation filter: 465–495 nm; Dichroic Mirror: 505 nm: Barrier Filter: 515–550 nm) was used. All images were acquired with a Nikon DS-Ri1 camera (Nikon UK) at an exposure time of 400 ms. Illumination intensity and camera settings, such as quality (16-bit), resolution (1280 × 1024), and colour (high contrast), were set and standardised to ensure that they were the same for all images, following previously described protocols^28^,^36^. Prior to any evaluation, bright field images were used to select sperm heads for analyses and only those sperm that presented the head attached to the tail, and did not overlap with other sperm, were selected. Fluorescent illumination was then used to outline the sperm heads through the region of interest manager (ROI) tool from ImageJ software (Version 1.46a; National Institutes of Health, Bethesda, MD, USA). For each slide, separate fields were randomly captured to image 100 sperm per sample. Mean PLCζ levels per sperm cell (arbitrary units) were quantified using Image J software. This mean value was derived from both sperm exhibiting PLCζ-staining and those that were devoid of staining. In addition, the proportion of sperm exhibiting PLCζ (in any localisation pattern), as well as the PLCζ localisation profile in each sperm cell, were evaluated following the procedure described by Grasa *et al.*^29^. Thus, PLCζ expression was assessed in each of the following eight localities: acrosomal (A); post-acrosomal (PA); equatorial (E); acrosomal and post-acrosomal (A + PA); acrosomal and equatorial (A + E); post-acrosomal and equatorial (PA + E); acrosomal, post-acrosomal and equatorial (A + PA + E); or ‘none’ indicating sperm totally devoid of PLCζ.

### Statistical analyses

Data were managed using Microsoft Excel 2010 (Microsoft Corp., Redmond, Washington, USA) and IBM SPSS 21.0 for Windows; IBM Corp., Chicago, Illinois, USA) and the two graphs were prepared with Origin Pro 8.0 software (OriginLab Corp., Northampton, Massachusetts, USA).

Three different statistical approaches were taken after first checking the data for normality (Shapiro-Wilk test) and homogeneity of variance (homoscedasticity; Levene test). Since some of the data (x) in proportions did not fit with parametric assumptions, they were transformed using arcsine root transformation (arcsin √x) and, when this transformation did not correct data normality and variance homogeneity, alternative non-parametric tests were used. Data for sperm motile counts were log-transformed prior to analysis. [log (x)].

The first statistical approach considered male age as a categorical factor. Thus, the effects of age and group (fertile controls and infertile patients) on proportions, total levels and localisation patterns of PLCζ, total motile count and the proportion of progressive motile sperm, were evaluated by two-way analysis of variance (ANOVA) followed by a post-hoc *t*-test with Bonferroni adjustment for pair-wise comparisons (the two factors used were the (i) age and (ii) the control/patient grouping). Since data for total proportions of sperm exhibiting PLCζ, and proportions of sperm with PLCζ localisation in E, A + PA and A + E did not match parametric assumptions, even after transformation, a two-way, non-parametric ANOVA for ranked data (Scheirer-Ray-Hare approach) was performed^58^. This approach is an extension of the Kruskal-Wallis test for two or more factors and calculates *P* values both for factors and their interaction by calculating an ‘H’ statistic. This H statistic results from dividing the sum of squares of a given factor (SS) by the mean squares (MS) and is then tested as a chi-square variable using the degrees of freedom pertaining to each factor (SS). The Mann-Whitney test was used to compare controls and patients regardless of age grouping, and to compare the two age groups regardless of whether data came from controls or patients.

The second approach considered male age as a continuous variable. In this case, correlation coefficients were calculated between male age, PLCζ parameters, motile count and the proportion of progressive motile sperm. These correlations were calculated with and without separating the statistical groups between controls and patients. Pearson correlations were used for these variables that required, or did not require, transformation, matched with parametric assumptions. In all other cases, Spearman correlations were carried out.

In the third approach, cases were first clustered via two-step cluster analysis using proportions, levels and localisation patterns as continuous variables. The log-likelihood was utilised as a measure for distance and the clustering was performed following Schwarz’s Bayesian Criterion (BIC). Descriptive statistics were calculated for the resulting clusters. The next step consisted of conducting logistic regression analyses using the number of clusters as the dependent variable and male age and grouping (controls vs. patients) as covariates. The method used was a forward stepwise protocol based on the likelihood ratio to determine the statistical significance of the factors as well as the order of importance. The probability-to-enter a variable into the model was 0.05 and the probability-to-remove was 0.10.

In all statistical tests, the significance level (two-tailed) was set at *P* ≤ 0.05. Data are expressed as mean ± standard error of the mean (SEM).

## Additional Information

**How to cite this article**: Yeste, M. *et al.* Does advancing male age influence the expression levels and localisation patterns of phospholipase C zeta (PLCζ) in human sperm? *Sci. Rep.* **6**, 27543; doi: 10.1038/srep27543 (2016).

## Figures and Tables

**Table 1:** Proportions (%) of sperm exhibiting PLCζ (mean ± SEM) in fertile controls and infertile patients in two age groups.

PLCζ localisation	Fertile Controls (n = 44)		Infertile Patients (n = 27)	
	<40 years	≥40 years	<40 years	≥40 years
A	1.4 ± 0.5	1.2 ± 0.6	2.4 ± 1.2	2.6 ± 1.2
PA	8.1 ± 2.1	8.0 ± 2.3	6.2 ± 2.0	7.6 ± 2.5
E	31.5 ± 5.3^a^	23.3 ± 6.2^a,b^	14.5 ± 3.9^b,c^	10.1 ± 4.3^c^
A + PA	0.9 ± 0.2	0.8 ± 0.6	2.1 ± 0.9	3.5 ± 1.8
A+ E	4.7 ± 1.8	1.9 ± 0.7	0.9 ± 0.5	1.1 ± 0.5
PA+ E	22.2 ± 4.4	29.5 ± 7.4	24.1 ± 5.8	25.9 ± 9.8
A + PA +E	14.8 ± 3.1	19.3 ± 5.7	12.0 ± 4.5	12.4 ± 5.2
None	16.3 ± 2.8^a^	16.0 ± 5.5^a^	37.8 ± 7.7^b^	36.7 ± 10.8^b^

Different superscripts (*a, b, c*) denote significant differences between columns within a given row. [Specific localisation patterns of PLCζ are referred to as: A = acrosomal; E = equatorial; None = absence of PLCζ; PA = post-acrosomal. ‘None’ refers to sperm that were completely devoid of PLCζ].

**Table 2:** Correlation coefficients between age and different PLCζ patterns in fertile controls, in infertile patients, and without separating these two groups (overall).

PLCζ localisation	Fertile Controls (n = 44)	Infertile Patients (n = 27)	Overall (n = 71)
% Sperm exhibiting PLCζ	0.02	0.05	0.01
Total levels of PLCζ	0.14	−0.06	−0.02
Localisation patterns (%)			
A	0.13	0.02	0.09
PA	0.19	−0.08	0.08
E	−0.13	−0.21	−0.15
A + PA	0.17	0.11	0.14
A+ E	−0.04	0.01	−0.04
PA+ E	0.10	−0.06	0.03
A + PA +E	0.05	0.05	0.02
None	−0.14	0.32	0.07

No significant correlations were observed in any of the cases evaluated. [Specific localisation patterns of PLCζ are referred to as: A = acrosomal; E = equatorial; None = absence of PLCζ; PA = post-acrosomal. ‘None’ refers to sperm that were completely devoid of PLCζ].

**Table 3:** Descriptive parameters (mean ± SEM) for the two clusters obtained following two-step cluster analysis using all sperm samples collectively.

PLCζ	Cluster 1	Cluster 2
N	47	24
% Sperm exhibiting PLCζ	88.9 ± 1.2	36.9 ± 5.5
Total levels of PLCζ	37.2 ± 2.3	22.6 ± 2.6
Localisation patterns (%)		
A	1.4 ± 0.4	2.4 ± 1.0
PA	7.4 ± 1.4	7.9 ± 1.9
E	29.3 ± 3.8	10.8 ± 2.9
A + PA	1.4 ± 0.5	1.3 ± 0.6
A+ E	3.5 ± 1.1	1.0 ± 0.4
PA+ E	29.9 ± 3.9	8.5 ± 1.8
A + PA +E	15.7 ± 2.6	10.6 ± 3.0
None	11.3 ± 1.1	52.7 ± 5.8

[Specific localisation patterns of PLCζ are referred to as: A = acrosomal; E = equatorial; None = absence of PLCζ; PA = post-acrosomal. ‘None’ refers to sperm that were completely devoid of PLCζ].

**Table 4:** Correlation coefficients between male age, % progressive motile sperm and total sperm motile count in fertile controls, infertile patients, and when both groups were analysed collectively (overall).

Parameter	Fertile Controls (n = 44)	Infertile Patients (n = 27)	Overall (n = 71)
% Progressive motile sperm	−0.31*	−0.29*	−0.34*
Total sperm motile count	−0.28*	−0.27*	−0.30*

Asterisk (*) denotes significant correlation (*P* < 0.05).
